# Health Care Professionals and Data Scientists’ Perspectives on a Machine Learning System to Anticipate and Manage the Risk of Decompensation From Patients With Heart Failure: Qualitative Interview Study

**DOI:** 10.2196/54990

**Published:** 2025-01-20

**Authors:** Joana Seringa, Anna Hirata, Ana Rita Pedro, Rui Santana, Teresa Magalhães

**Affiliations:** 1 NOVA National School of Public Health, Public Health Research Centre, Comprehensive Health Research Center, NOVA University Lisbon Lisbon Portugal; 2 NOVA National School of Public Health, NOVA University Lisbon Lisbon Portugal

**Keywords:** heart failure, machine learning system, decompensation, qualitative research, cardiovascular diseases, heart failure management, interview

## Abstract

**Background:**

Heart failure (HF) is a significant global health problem, affecting approximately 64.34 million people worldwide. The worsening of HF, also known as HF decompensation, is a major factor behind hospitalizations, contributing to substantial health care costs related to this condition.

**Objective:**

This study aimed to explore the perspectives of health care professionals and data scientists regarding the relevance, challenges, and potential benefits of using machine learning (ML) models to predict decompensation from patients with HF.

**Methods:**

A total of 13 individual, semistructured, qualitative interviews were conducted in Portugal between October 31, 2022, and June 23, 2023. Participants represented different health care specialties and were selected from different contexts and regions of the country to ensure a comprehensive understanding of the topic. Data saturation was determined as the point at which no new themes emerged from participants’ perspectives, ensuring a sufficient sample size for analysis. The interviews were audio recorded, transcribed, and analyzed using MAXQDA (VERBI Software GmbH) through a reflexive thematic analysis. Two researchers (JS and AH) coded the interviews to ensure the consistency of the codes. Ethical approval was granted by the NOVA National School of Public Health ethics committee (CEENSP 14/2022), and informed consent was obtained from all participants.

**Results:**

The participants recognized the potential benefits of ML models for early detection, risk stratification, and personalized care of patients with HF. The importance of selecting appropriate variables for model development, such as rapid weight gain and symptoms, was emphasized. The use of wearables for recording vital signs was considered necessary, although challenges related to adoption among older patients were identified. Risk stratification emerged as a crucial aspect, with the model needing to identify patients at high-, medium-, and low-risk levels. Participants emphasized the need for a response model involving health care professionals to validate ML-generated alerts and determine appropriate interventions.

**Conclusions:**

The study’s findings highlight ML models’ potential benefits and challenges for predicting HF decompensation. The relevance of ML models for improving patient outcomes, reducing health care costs, and promoting patient engagement in disease management is highlighted. Adequate variable selection, risk stratification, and response models were identified as essential components for the effective implementation of ML models in health care. In addition, the study identified technical, regulatory and ethical, and adoption and acceptance challenges that need to be overcome for the successful integration of ML models into clinical workflows. Interpretation of the findings suggests that future research should focus on more extensive and diverse samples, incorporate the patient perspective, and explore the impact of ML models on patient outcomes and personalized care in HF management. Incorporation of this study’s findings into practice is expected to contribute to developing and implementing ML-based predictive models that positively impact HF management.

## Introduction

Heart failure (HF) is a complex clinical syndrome characterized by frequent and rapid decompensation; even long-term, stable patients with HF might rapidly deteriorate in days or hours [1]. HF decompensation is a major cause of hospitalization in patients with HF, negatively influencing prognosis [1], representing the highest share of health care costs for this disease [2].

Several factors can precipitate HF decompensation, including respiratory infections, noncompliance with dietary recommendations or pharmacological treatment, and atrial fibrillation [2]. Early detection of HF decompensation is crucial to allow timely intervention and to prevent hospitalizations and increased morbidity [3].

Studies suggest that telemonitoring systems and predictive models for clinical support and patient empowerment may improve HF management [4]. Based on telemonitored data, artificial intelligence (AI) techniques have been increasingly used to predict and analyze HF decompensation events. AI and machine learning (ML) algorithms have the power to assimilate and integrate multidimensional, multimodal data and create accurate prediction models. The application of AI/ML algorithms could potentially improve workflow and outcomes for patients with HF due to the ever-growing data, particularly time series data gathered via remote monitoring [5]. Furthermore, there have been other health conditions in which AI models have been successfully developed for early detection and screening. A study published in the scientific journal *Nature* showcased a model that surpassed radiologists’ performance in interpreting mammograms [6], while another study introduced an ML model that reliably predicts the onset of diabetes [7].

However, there is a limited understanding of health care professionals’ perspectives on using such systems and the specific considerations necessary for designing an ML model to be integrated into clinical practice. To bridge this knowledge gap, we conducted a semistructured interview study involving health care professionals and data scientists with profound expertise in health care. The primary objective of this study was to explore their viewpoints concerning an ML system tailored for the early detection and management of HF decompensation. Additionally, this study sought to provide the experts’ perceptions on developing crucial features in the ML system, identify potential implementation challenges, and establish design principles to enhance overall system efficiency and effectiveness.

## Methods

### Study Design

A qualitative research design was used, using individual semistructured interviews to explore the perspectives of health care professionals and data scientists regarding the relevance, challenges, and potential benefits of using ML models to predict decompensation in patients with HF.

### Eligibility Criteria

Health care professionals working with patients with HF and data scientists with expertise in the health care field were considered eligible to participate in this study.

### Recruitment Strategies

Participants were selected through intentional sampling to ensure the representation of diverse perspectives and expertise in HF and health care data management.

Potential participants were identified based on their expertise in the field. With prior authorization from the experts, key contacts from the researchers’ network shared their email addresses with the primary researcher, facilitating direct communication.

The primary researcher emailed potential participants and invited them to a semistructured interview. This email included attachments to the interview guide and a consent form. The participants were required to provide written consent to participate in this study by signing the consent form.

### Participants

A total of 13 participants, representing a diverse range of health care professionals and data science experts, were interviewed for this study. The participants recruited were primary care physicians (n=1), cardiologists (n=3), internal medicine physicians (n=3), nurses (n=3), and data scientists (n=3). The composition of this poll of experts aimed to capture the perspective of the health care professionals who usually provide care to patients with HF and the technical perspective of the data scientists. Geographically, participants were in different areas, with 2 participants based in the north of Portugal, 8 participants in the Lisbon area and its surroundings, 2 participants in the south, and 1 participant working outside of Portugal. The diversity of locations is especially relevant to guarantee the results are not biased by the local context.

The sample size of 13 participants was determined based on data saturation, where no new themes emerged from participants’ perspectives, aligning with a recent systematic review’s findings indicating that saturation can be achieved within a narrow range of interviews (9-17) in qualitative research [8].

### Data Collection

The primary researcher conducted face-to-face and online semistructured interviews in Portuguese, using an interview guide (Multimedia Appendix 1) developed by the authors after the participants provided written informed consent to participate. All interviews were conducted in private rooms by a single researcher and were audio recorded to ensure accurate data capture.

The interview guide explored the participants’ perspectives on an ML model for predicting HF decompensation. Open-ended questions were included in the interview guide, allowing participants to express their thoughts and opinions freely.

Before the formal data collection, an initial interview was conducted with an internal physician to test and adjust the interview guide, ensuring its relevance and appropriateness; this first interview was not included in the subsequent analysis.

### Data Analysis

Each interview lasted, on average, 31 (range 15-48) minutes. The interviews were audio recorded, transcribed verbatim, and assessed for accuracy.

The participant’s data were analyzed using interactive and inductive processes following a thematic analysis approach as proposed by Braun and Clarke [9], since the purpose of the research was explanatory and intended to generate new knowledge. The data analysis was conducted in the following phases: (1) familiarizing with the dataset, (2) generating initial codes, (3) searching for themes, (4) reviewing the themes, (5) defining and naming the themes, and (6) producing the report [9].

In the first phase, two authors engaged with the dataset by reviewing the interviews multiple times to understand the content’s depth, identifying key expressions, and formulating and validating meanings through discussions. In the second phase, the MAXQDA (VERBI Software GmbH) was used to generate initial codes, allowing for simplified data analysis and management. Subsequently, codes were categorized into overarching themes.

During the third phase, the two authors (JS and AH) systematically identified and organized themes into clusters and categories. In the fourth phase, a third author (ARP) with expertise in qualitative research reviewed the findings to enhance the credibility of the analysis. The author provided feedback on the analytical process and the developing themes, facilitating their refinement.

In the fifth phase, the authors refined and defined the themes by re-examining the dataset and developing a comprehensive description of the themes. In the sixth phase, excerpts of the interview, derived from coding, were translated from Portuguese to English and further edited for clarity, resulting in the report of the analyzed dataset.

### Researchers’ Reflexivity

In reflexive thematic analysis, the researchers incorporate their knowledge and experiences into their analytical practice [10]. It is important to highlight that all the authors, as health care researchers, have engaged with patients diagnosed with chronic diseases, including HF, and are particularly focused on understanding how digital health can contribute to better management of these conditions. Therefore, our values and assumptions might have influenced the analysis produced. However, efforts were made to minimize this influence through rigorous data analysis and researchers’ discussions.

### Ethical Considerations

The research was conducted per the Helsinki Declaration (World Medical Association, 2018), and written informed consent was obtained from every participant. All data collected during the interviews were anonymized to ensure participant confidentiality. Only the primary researcher has access to the original recordings, which are securely stored and protected. To ensure anonymity, all participants were assigned unique codes, and personal identifiers were removed during data transcription to safeguard the privacy of participants. No participant received any type of compensation for taking part in this study. The study was approved by the NOVA National School of Public Health research ethics committee (CEENSP no 14/2022).

## Results

### Overview

Saturation was achieved with the seventh interview, and 6 participants were then interviewed to confirm thematic saturation. Four themes or categories emerged from the analysis: “Machine learning model relevance,” “Variables collection and system features,” “Risk levels and their management,” and “Challenges and potential solutions for implementation.”

### Thematic Content

**Figure 1:**
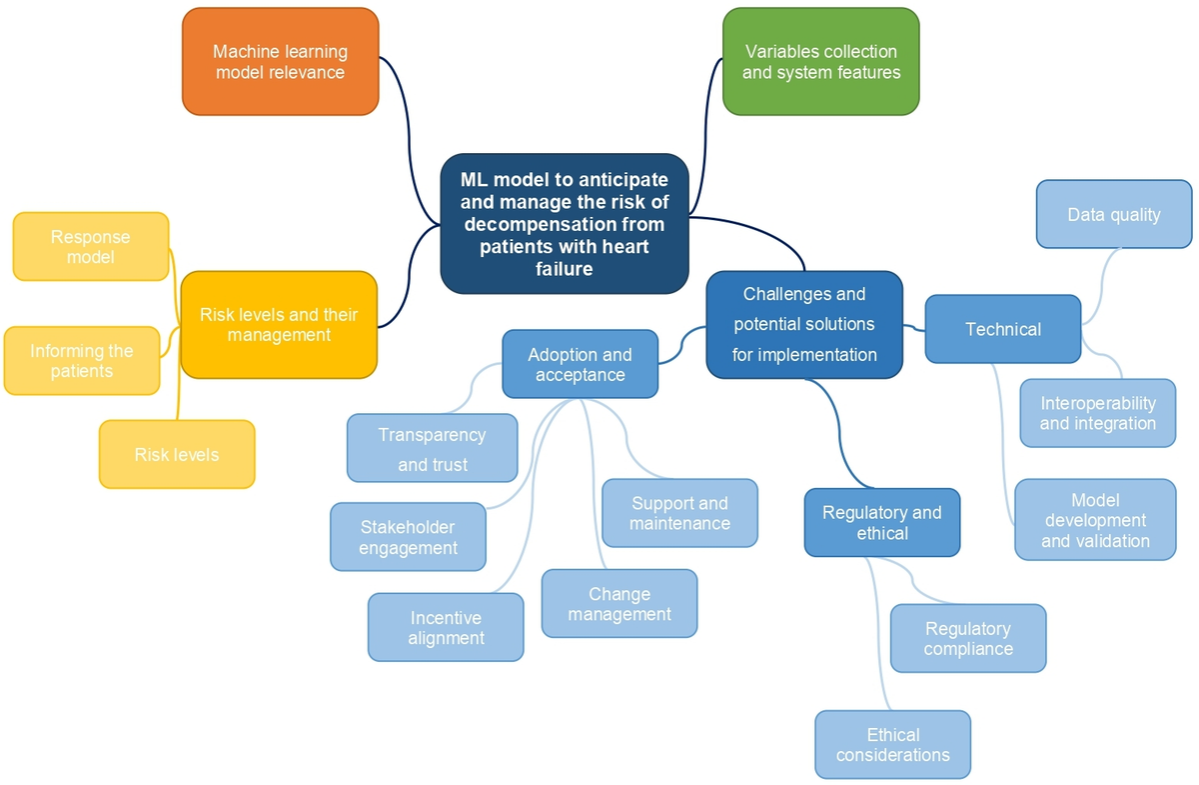
Subgroups and themes or categories identified in the analysis. ML: machine learning.

Subgroups and themes or categories identified in the analysis of health care professionals and data scientists’ perspectives of an ML model to predict the risk of decompensation from patients diagnosed with HF are illustrated in Figure 1. Excerpts of the interview presented in the results were extracted from the coding analysis, and the themes and subgroups resulting from the qualitative coding are reported below.

### ML Model Relevance

Most participants highlighted the potential benefits of using an ML model to predict decompensation from patients with HF. These benefits included identifying patients at risk of decompensation earlier, allowing for more personalized care, improving patient outcomes, and reducing health care costs.

Is a very relevant thing, and early detection of decompensation is something that allows for reducing the number of hospitalizations, which is fundamental. Not only for the patient’s quality of life but also for the burden on the National Health Service.

Cardiologist 2

Regarding models in general, predictive models, I think they are well received by health professionals...There is a clear notion that we are evolving toward an increasingly personalized medicine. We also know that today, waiting for these changes that start as subclinical to become clinical so that professionals or patients then feel that they exist; we are losing, in quotes, time, where intervention could be done earlier and more deleterious outcomes could be avoided, which in the case of heart failure in particular, the one always intended to be achieved is to avoid hospitalizations. Since any additional hospitalization in these patients has a worse prognosis in the natural history of the disease, the earlier we can adjust therapy so that no decompensation of heart failure leads to hospitalization, not only from the individual’s perspective but also from the institution’s perspective, avoiding pressure on emergency services and hospitals in general.

Internal medicine physician 2

Participants also considered the model could contribute to better management of the disease by the patient.

For me, the great advantage of digital technology in heart failure, for health professionals, is that it allows us to start preventing hospitalizations earlier. I think that for the patient, even those who are more reluctant to adhere to therapy because in heart failure, therapeutic adherence is also a problem; it gives them a certain empowerment over the disease......So, I think it’s a win-win for both sides.

Nurse 2

### Variables Collection and System Features

Participants discussed the importance of identifying the correct variables for the ML model. Some variables mentioned include rapid weight gain and symptoms such as tiredness, shortness of breath, and swelling of the ankles (see Textbox 1 for a complete list of variables). Participants noted that selecting variables should be evidence-based and consider the complexity of HF management.

Weight gain of more than 2kg in 3 days; increase the number of pillows, the patient who sleeps with one pillow to two or three, or to sleep sitting up; increased edema of the lower limbs; increased coughing at night; finally, a feeling of worsening tiredness for minor efforts.

Internal medicine physician 1

**List of variables mentioned by the participants.**

- Patient characterization: sex, age, comorbidities, and previous hospitalizations
- Heart failure characterization: New York Heart Association Classification and left ventricular ejection fraction
- Vital signs: weight, height, systolic blood pressure, diastolic blood pressure, heart rate, and blood oxygen saturation
- Laboratory results: creatinine, glomerular filtration rate based on the chronic kidney disease epidemiology collaboration equation, hemoglobin, hematocrit, urea, N-terminal pro b-type natriuretic peptide (NT-Pro BNP), ferritin, sodium, potassium, iron (fix capacity, iron), saturation (from transferrin), natriuretic peptide (BNP), and iron
- Prescribed medication: furosemide, bisoprolol, dapagliflozin, spironolactone, sacubitril, valsartan, carvedilol, lisinopril, nebivolol, ramipril, perindopril, torsemide, enalapril, empagliflozin, captopril, eplerenone, propranolol, atenolol, timolol, trandolapril, fosinopril, metoprolol, and sotalol

The collection of vital signs through wearables was deemed relevant by the participants.

I think asking someone every day, and even if it’s every week, to record their weight, blood pressure, and frequency is tough. I don’t think that’s feasible in the long run. So the simpler, the better, and there, the devices are clearly in the way.

Internal medicine physician 3

However, as HF is prevalent among older people, using such devices was considered a challenge by some interviewees.

Most people with HF are elderly, and the likelihood of using wearables will be low.

Primary care physician 1

Interviewers also highlighted the need to select the population to use these tools properly.

Telemonitoring has two secrets. One of them is, to be cost-effective, the population you choose; I don’t think it’s a technology applied to all patients.

Cardiologist 1

### Risk Levels and Their Management

#### Risk Levels

Participants emphasized the importance of developing an ML model capable of stratifying patients into distinct risk levels (high, medium, and low risk of decompensation), considering the patient’s HF journey stage. This approach would facilitate health care professionals to provide more targeted interventions.

The literature in cardiovascular patients structures patients into three levels, possibly four. Roughly speaking, therefore, that is low risk, moderate risk, high risk, and, in some situations, there is a very high or very high-risk level. In the case of heart failure, risk stratification will also depend on the type of patient, namely the stage of their heart failure journey......three levels, roughly speaking, sounds good, I think it’s interesting in your work that you can adjust this risk stratification also to the stage of the heart failure patient journey.

Internal medicine physician 2

#### Response Model

Several participants discussed the importance of having a response model to validate the alerts generated by the ML model. They noted that the validation process should involve a health care professional, such as a nurse or a physician, who could assess the patient’s condition and determine the appropriate response.

On the first level, the ideal situation would be to activate a call from a specialist heart failure nurse who would help the patient identify what they are doing right and what they are doing wrong. At an intermediate level...the structure would be the equivalent of an open consultation, a day hospital...a medical team. They may go through a nursing assessment first while consulting with the heart failure medical team. In the red level, I think the medical team must be activated to help decide what is going on and how the patient will be assessed.

Internal medicine physician 3

#### Informing the Patients

Participants discussed the potential benefits and challenges of providing alerts to patients identified as high-risk by the ML model. They noted that alerts could help patients take proactive steps to manage their condition, but there were concerns about patient anxiety.

When the information is to be given to the patient, either because it is considered that it has to be given, or because the patient has asked for this information, it cannot just be “you have an alert,” there has to be an explanation. There has to be an explanation and especially a recommendation of what to do next, because otherwise, it creates a level of anxiety, “you have a change in your oxygen (levels)” (alert) and that’s it. It would be a bit distressing for anyone.

Internal medicine physician 2

### Challenges and Potential Solutions for Implementation

The participants identified several challenges regarding implementing the ML model, along with suggestions to address the identified challenges. They can be grouped into the following categories.

#### Technical Challenges

##### Data Quality

Participants emphasized the importance of obtaining high-quality and reliable health care data for training accurate ML models. They noted that health care data can often be messy, incomplete, or contain errors, posing challenges in data preprocessing and cleaning.

There are other issues, not only of having rich data set but also types of quality of data sets, trying to understand how that type of data was collected, how that data was treated, how they were coded. For example, whether ICD 10 or 11 (is used), how are the coding practices like, the standard practice in terms of coding...all of this is necessary to understand the rules inherent to the type of data we have.

Data scientist 1

##### Model Development and Validation

Participants highlighted that designing and developing ML models suitable for health care applications requires domain expertise. It involves selecting appropriate algorithms, fine-tuning models for accuracy, interpretability, and generalizability, and conducting robust testing for model validation.

##### Interoperability and Integration

The participants mentioned that integrating ML models into existing health care systems and workflows can be challenging. They pointed out issues related to interoperability, compatibility with different data formats and systems, and the need for seamless integration with electronic health records and other health care software.

All of this (defining which data to feed into an ML model, EHRs, prescription drugs) also influences the cardiac care...and of course...take into account that many of these systems do not talk to each other, so there is no interoperability of systems, and this makes it difficult from the outset. There is, therefore, work to do beyond quality (assurance) to make all the data talk to each other, trying to see how possible it is to go to the individual level, that is, what data do you have and how can you have as much information as possible at the individual level...But in any case, interoperability here will be very crucial for how fast you can have the data ready for the model, even before deciding which model will be the best for this type of function.

Data scientist 2

#### Regulatory and Ethical Challenges

##### Regulatory Compliance

Compliance with regulatory frameworks, such as the General Data Protection Regulation in the European Union, was identified as a significant challenge in implementing ML models in health care. Participants stressed the importance of adhering to legal and ethical guidelines regarding data use, consent, and transparency, mainly due to the sensitive nature of health care data, such as patient records. Ensuring patient privacy and data security while using ML models posed notable challenges.

Without a doubt, there is a need to ensure that there is some guarantee here in terms of privacy and data security; this becomes immediate when you are joining many sources of data.

Data scientist 2

##### Ethical Considerations

The participants emphasized the need to develop and deploy ML models with ethical considerations. They underscored the importance of avoiding bias, ensuring fairness and equity in health care outcomes, and maintaining transparency in decision-making processes.

The real bias is systemic and must be controlled. Other studies (demonstrate the impact of) minorities who were underrepresented; then obviously when calculating the scores, they were underestimated, and the system continued as it always has, leaving those people on the sidelines.

Data scientist 1

There is another important issue, which is the representativeness and inclusivity within the data set. Many problems have happened because of the type of data and the conclusions reached that sometimes are not fair, (known as) algorithm justice.

Data scientist 1

These aspects were identified as crucial for building trust among health care professionals and patients.

#### Adoption and Acceptance Challenges

##### Transparency and Trust

As referred before, participants highlighted the importance of transparency in the ML model to gain the trust of health care professionals and patients, favoring the choice of models that have higher explainability, which refers to a characteristic of an AI-driven system that allows understanding and interpretation of the decisions and predictions made by an AI system [11].

Ensuring transparency in the decision-making process was perceived as crucial to fostering trust among stakeholders, as it allows them to understand the underlying factors and reasoning behind the ML model’s recommendations or predictions.

As you build a model, there is a need to keep those metrics (sensitivity, specificity, therefore, recall, precision, F-score, accuracy) in mind and to perform a primary assessment of those metrics. Another important part that has been talked about more in the last few years and will involve both patients and clinicians is the technical issue of explainability—the idea that AI models can’t just be a black box. If we want some form of engagement, some participation from both doctors and patients, and especially trust at the health care system level, as patients and clinical decisions are involved, the system should be something that we have to be able to explain to both stakeholders and patients and doctors.

Data scientist 1

##### Stakeholder Engagement

Engaging health care professionals, including physicians, nurses, and administrators, in designing and implementing ML models was highlighted as essential for successful adoption. Participants stressed the significance of addressing their concerns, providing training, and demonstrating the benefits of ML models to gain their support and involvement.

I think our role has to be to encourage through the constant involvement of the professional in the development of the model and implementation, as well as the validation process and everything, but also to demonstrate how those models are not the panacea.

Data scientist 2

##### Change Management

Participants acknowledged that implementing ML models might change health care organizations’ workflows, processes, and roles. Additionally, participants expressed concerns that ML models might create an increased workload for health care professionals. They emphasized the need for careful planning and allocation of resources to ensure that the integration of ML models into clinical workflows does not burden the health care workforce.

And the machine learning model was a part that, according to the set of information, whether it is from the electronic medical record, such as comorbidities, etc., whether it is collected daily, we have a model here that basically gives us alert levels, which can be, for example, three alert levels, low, medium, high, etc. and act only on those that are medium or high, so that we do not have too many false positives.

Cardiologist 3

##### Incentive Alignment

Participants raised the issue of financial incentives in health care, particularly the existing payment structure that often rewards hospitals based on the activity (number of admissions) rather than patient outcomes. They acknowledged that highly effective ML models capable of accurately predicting admissions might be challenging to implement due to misaligned incentives.

Today, the incentive system in virtually all health care systems is based on procedures, it is based on records made, compliance with a process defined as correct, or the number of surgeries or consultations carried out. So, that’s how productivity is measured, and compensation is defined, and that is problematic because the system is conditioned to have a short-term perspective. For example, there is a development of a model that manages to reduce hospitalizations by 50%. But the hospital, like everywhere else in Portugal, only gets reimbursed for the number of hospitalized patients. And the more seriously ill the patients are, the more they may be paid. You run the risk that the hospital thinks your model is very good, but in terms of incentives gained, it doesn’t make sense to be applied.

Data scientist 3

##### Support and Maintenance

Participants stressed the importance of having dedicated resources and personnel to provide ongoing support and address the doubts and concerns of health care professionals regarding the ML model.

In addition, the need for a designated team responsible for the maintenance and upkeep of the ML model was identified as crucial for ensuring its continuous performance, addressing any technical issues, and incorporating updates or improvements as needed.

We don’t have medical information officers...for software. I published an article with colleagues about mental health professionals’ perceptions of digital health tools, whether apps, websites, etc. One of the biggest problems was that the professionals did not know when to use these apps; they did not have guidelines or recognition from the societies and professional groups they belonged to. It was also noted that there was no contact and no use of those applications—when questioned about the support given by the tool developers, many participants either responded that they did not know or did not answer.

Data scientist 3

## Discussion

### Principal Findings

The findings of this study have several implications for the practical application of ML models in the early detection and management of HF decompensation. The identified themes and subgroups provide insights into critical considerations that health care professionals and data scientists consider relevant when developing and implementing ML models for predicting the risk of decompensation in patients with HF.

The participants recognized the potential benefits of using ML models for the early identification of patients at risk of decompensation, leading to improved patient outcomes and reduced health care costs, a notion that aligns with the findings from previous research [4,12-14].

The emphasis on the importance of early detection is particularly noteworthy, as it aligns with the evolving trend toward personalized care. The ability to intervene before subclinical changes become clinical can significantly impact patient outcomes, emphasizing the critical role of ML models in leading to timely intervention and avoiding hospitalizations [13,15]. This perception was echoed by health care professionals from various domains, that is, cardiologists, internal medicine physicians, and nurses.

The selection of appropriate variables is crucial for accurately predicting HF decompensation using ML models. The participants emphasized the importance of evidence-based variable selection and the consideration of the complexity of HF management. Variables such as rapid weight gain and reported symptoms such as orthopnea and swelling of lower limbs were mentioned as potentially relevant factors for inclusion in the ML models, which aligns with previous studies that identified these as significant predictors of cardiac decompensation events in patients with HF [13].

Furthermore, the use of wearables for collecting vital signs was seen as relevant, but the challenges related to adoption among older patients must be addressed. Additionally, the importance of adequate population selection to ensure cost-effectiveness was highlighted. These findings align with the current literature that acknowledges the potential benefits of the use of wearables but identifies a set of barriers that need to be addressed for further implementation of these devices into clinical practice [16].

Participants highlighted the importance of ML models identifying different risk levels for patients with HF, considering the patient’s HF journey stage [17,18]. By accurate risk stratification, health care professionals can prioritize their interventions and provide more targeted care to those at higher risk [19,20].

It was also identified the relevance of ML models being designed to provide risk stratification outputs that are easily interpretable and actionable by health care professionals. This may involve the development of user-friendly interfaces or decision support systems that show risk levels clearly and intuitively. This perspective aligns with the recognized need for interpretable ML models, as interpretable models enable users to examine, comprehend, troubleshoot, and potentially enhance ML systems. By clarifying the logic behind predictions, interpretable ML systems provide users with justifications for accepting or rejecting predictions and recommendations [21].

Additionally, the participants emphasized the need for a response model to validate the alerts generated by the ML models. The involvement of health care professionals, such as nurses or physicians, in assessing patients’ conditions and determining appropriate responses is crucial for effectively managing decompensation.

The study identified several challenges in implementing ML models for predicting HF decompensation and potential solutions. These can be grouped into three categories: technical, regulatory and ethical, and adoption and acceptance challenges, which are aligned with this literature [22,23].

The importance of high-quality and reliable health care data was recognized, with an acknowledgment of the complexities involved in data preprocessing and cleaning, as already addressed in the literature [24]. The need for domain expertise in designing and developing ML models was emphasized, along with challenges related to interoperability and integration with existing health care systems. The existence of proposed frameworks to guide the development and integration of ML models in health care, such as the one developed by Assadi et al [25], frames the findings of this study within this literature.

Compliance with regulatory frameworks, particularly regarding data protection, was identified as a significant challenge. Ethical considerations, including avoiding bias and ensuring fairness and transparency, were highlighted as crucial for building trust among health care professionals and patients, which is in line with what is stated by the European Parliament [26] and recent literature [27].

Transparency and trust were emphasized as critical factors for the successful adoption of ML models [28]. Stakeholder engagement, particularly involving health care professionals, was recognized as essential, along with considerations for change management to avoid disrupting existing workflows, which had already been identified in a similar study [29].

The presence of misaligned financial incentives within the health care system was recognized as a potential obstacle to the integration of ML models. This underscores the importance of establishing a successful reimbursement strategy to align interests among stakeholders, thereby guiding the application of value-based AI in health care, as payment strategies impact utilization, cost, and quality of care across the clinical use cases for AI [30]. A recent paper in *NPJ Digital Medicine* expands into the discussion on the appropriate method of reimbursement for AI systems in health care [31].

The importance of ongoing support, addressing the concerns of health care professionals, and having a dedicated team for the maintenance of the ML model was also recognized as relevant for successfully implementing these models into practice [32].

In general, this study’s findings emphasize the potential of ML models in improving the early detection and management of HF decompensation. Considering the implications for practice outlined above, this study is expected to contribute to developing and implementing ML models that positively impact patient outcomes, health care costs [33], and the delivery of personalized care in HF management.

In addition, this study highlighted the synergy between the perspectives of health care professionals and data scientists not only enhances the accuracy and effectiveness of ML models but also fosters a holistic approach that considers both the clinical and technical facets, promoting the successful integration of these models into health care practices. From the health care professionals’ perspective, there is a strong emphasis on evidence-based variable selection, drawing on clinical expertise to identify relevant indicators for predicting decompensation in patients with HF. Their insights into the nuanced aspects of patient care, risk stratification, and the practical implications of ML models contribute crucially to the model’s alignment with real-world clinical workflows.

On the other side, data scientists bring technical expertise to the table, highlighting challenges related to data quality, model development, and interoperability. They underscore the importance of domain knowledge in ML model development, recognizing the complexities involved in designing accurate, interpretable, and generalizable models for health care applications. Data scientists also stress ethical considerations, algorithmic fairness, and the need for transparent decision-making processes, emphasizing the broader societal impact of ML models in health care. In short, close collaboration between these two professional domains is paramount [25].

Future research should address these concerns and evaluate the effectiveness of ML models in improving patient outcomes.

### Strengths and Limitations

The findings of this study provide relevant insights into health care professionals’ and data scientists’ perspectives on using ML models for predicting HF decompensation. However, it is essential to consider the strengths and limitations of the study methodology and findings.

Regarding the strengths, using a qualitative research design allowed for an in-depth exploration of health care professionals’ and data scientists’ perspectives on ML models for HF decompensation prediction. The semistructured interviews provided rich and detailed data, enabling a comprehensive analysis of themes and subgroups. In addition, the thematic analysis approach used in this study, following the guidelines proposed by Braun and Clarke [9], provided a systematic and rigorous process for analyzing the data. Two researchers were involved in the analysis process, enhancing the credibility and reliability of the findings.

The diverse range of health care professionals, including a primary care physician, cardiologists, internal medicine physicians, nurses, and data scientists, and the high level of experience from the participants are also strengths of the study, enhancing the validity and generalizability of the findings. In addition, the fact that participants were from different contexts and regions across the country is also a positive point, reducing the influence of the local context on the overall results.

The determination of data saturation, where no new themes emerged from participants’ perspectives, ensured sufficient sample size was achieved to capture the breadth and depth of the participants’ experiences and opinions.

Considering the study’s limitations, we emphasize that, although data saturation was reached, the sample size of 13 participants may be considered relatively small. The findings may not fully represent the perspectives of all health care professionals involved in HF management and data scientists’ considerations on this specific issue.

In addition, data were collected solely through semistructured interviews. While interviews provide rich qualitative data, multiple data collection methods, such as surveys or observation, could have provided a more comprehensive understanding of the topic.

As with any qualitative study, there is a potential for researcher bias during data collection, analysis, and interpretation. The researchers’ perspective may have influenced the findings. However, efforts were made to minimize this influence through rigorous data analysis and researchers’ discussions.

In addition, another significant limitation of this study is the potential loss of information during the translation process of the interviews from Portuguese to English. Language translation can be complex, and nuances, cultural references, and contextual information may not always be accurately expressed in the translated version. This could lead to the loss of relevant insights and subtle meanings expressed by the participants in their original language. To mitigate this limitation, cross-validation of translations and back-translation were used to ensure the accuracy of the translated data.

Furthermore, the patients’ perspectives were not included in this study. Incorporating patient viewpoints would have provided a more holistic understanding of the potential benefits and challenges associated with ML models for HF decompensation prediction.

Future research should address these limitations by including more extensive and diverse samples, incorporating multiple data collection methods, and considering patients’ perspectives.

### Conclusions

This study explored health care professionals and data scientists’ perspectives on using ML models to predict HF decompensation. The findings provide important insights into the potential benefits, challenges, and considerations associated with implementing ML models in HF management.

The study highlighted several key themes, including the relevance of ML models in identifying patients at risk of decompensation earlier, the importance of selecting appropriate variables for model development, the need for risk stratification and response models, and the challenges and potential solutions for implementing ML models in health care settings. These findings have implications for practice in HF management and the development of effective ML-based predictive models.

Future research should address the limitations of this study, such as expanding the sample size and including patients’ viewpoints. Additional research areas include evaluating ML models’ impact on patient outcomes, health care costs, and the delivery of personalized care.

The incorporation of this study’s findings into practice is expected to contribute to the development and implementation of ML models that can potentially improve the early detection and management of HF decompensation, enhancing patient outcomes and reducing health care costs.

## Appendix

Multimedia Appendix 1 Guide for semistructured interviews.

## Abbreviations

AI: artificial intelligence
HF: heart failure
ML: machine learning

## Data Availability

The datasets generated during or analyzed during this study are available from the corresponding author upon reasonable request.
