# Supplementary material for: Health Care Professionals and Data Scientists’ Perspectives on a Machine Learning System to Anticipate and Manage the Risk of Decompensation From Patients With Heart Failure: Qualitative Interview Study
Source: J Med Internet Res. 2025 Jan 20;27:e54990. doi: 10.2196/54990 (PMC11791461; doi:10.2196/54990)
Supplement: Multimedia Appendix 1 [file jmir_v27i1e54990_app1.pdf]

Guide for semi-structured interviews:

Participating group	Theme	Main questions
Healthcare professionals	Understand the perspective of healthcare professionals regarding the relevance of the model	- What is your perspective on developing a machine learning-based model to prevent and manage urgent hospital admissions for people with heart failure?<br>- To what extent do you consider that the integration of information from wearables into the patient's electronic clinical record could contribute to the prevention of urgent hospital admissions for patients with heart failure?
Healthcare professionals	Identification of the main variables to be collected	- What do you consider to be the main variables (e.g.: vital signs and self-reported information) to be collected to anticipate urgent hospital admission for patients with heart failure?
Healthcare professionals	Definition of risk levels and their management	- What is your perspective regarding the creation of 3 risk levels of urgent hospital admission for people with heart failure: “priority”, “very priority” and “urgent”?<br>- How far in advance should an intervention be carried out by the healthcare professional, given each of the identified risk levels, to be effective in preventing an urgent admission?<br>- In your opinion, what is the best way to communicate the level of risk of an urgent hospital admission to a healthcare professional?<br>- What is your perspective regarding the automatic scheduling of a consultation (remote or in-person) in the information system for the person who presents one of the identified risk levels?<br>- Do you consider that the patient should also

		have access to this information (risk of urgent hospital admission)? In what way?

**Participating group**	**Theme**	**Main questions**
Data scientists	Model development and implementation	- In your opinion, what are the main technical concerns related to implementing this model based on machine learning?<br>- How can technical constraints be mitigated?<br>- In your opinion, what are the main ethical concerns related to the implementation of this model based on machine learning?<br>- How can ethical constraints be mitigated?
